# Mathematical modeling and machine learning-based optimization for enhancing biofiltration efficiency of volatile organic compounds

**DOI:** 10.1038/s41598-024-65153-7

**Published:** 2024-07-23

**Authors:** Muhammad Sulaiman, Osamah Ibrahim Khalaf, Naveed Ahmad Khan, Fahad Sameer Alshammari, Habib Hamam

**Affiliations:** 1 Department of Mathematics, Abdul Wali Khan University, 23200 Mardan, Pakistan; 2 Department of Solar, Al-Nahrain Research Center for Renewable Energy, Al-Nahrain University, Jadriya, Baghdad, Iraq; 3 School of Information Technology and Systems, University of Canberra, Canberra, ACT Australia; 4 Academy of Mathematics and Systems Science, Chinese Academy of Sciences, Beijing, China; 5 Department of Mathematics, College of Science and Humanities in Alkharj, Prince Sattam bin Abdulaziz University, Al-Kharj, 11942 Saudi Arabia; 6 Faculty of Engineering, Université de Moncton, Moncton, NB E1A3E9 Canada; 7 Hodmas University College, Taleh Area Mogadishu, Somalia; 8 Bridges for Academic Excellence, Tunis Centre-Ville, Tunisia; 9 School of Electrical Engineering, University of Johannesburg, Johannesburg, 2006 South Africa

**Keywords:** Mathematical modeling, Reaction mechanism, Volatile organic compounds, Michaelis-Menten kinetics, Supervised machine learning, Elman neural networks, Optimization, Artificial Intelligence, Engineering, Mathematics and computing

## Abstract

Biofiltration is a method of pollution management that utilizes a bioreactor containing live material to absorb and destroy pollutants biologically. In this paper, we investigate mathematical models of biofiltration for mixing volatile organic compounds (VOCs) for instance hydrophilic (methanol) and hydrophobic ($\alpha$-pinene). The system of nonlinear diffusion equations describes the Michaelis-Menten kinetics of the enzymic chemical reaction. These models represent the chemical oxidation in the gas phase and mass transmission within the air-biofilm junction. Furthermore, for the numerical study of the saturation of $\alpha$-pinene and methanol in the biofilm and gas state, we have developed an efficient supervised machine learning algorithm based on the architecture of Elman neural networks (ENN). Moreover, the Levenberg-Marquardt (LM) optimization paradigm is used to find the parameters/ neurons involved in the ENN architecture. The approximation to a solutions found by the ENN-LM technique for methanol saturation and $\alpha$-pinene under variations in different physical parameters are allegorized with the numerical results computed by state-of-the-art techniques. The graphical and statistical illustration of indications of performance relative to the terms of absolute errors, mean absolute deviations, computational complexity, and mean square error validates that our results perfectly describe the real-life situation and can further be used for problems arising in chemical engineering.

## Introduction

The category of carbon-based molecules known as volatile organic compounds (VOCs) are characterized by their propensity for rapid evaporation and dynamic behavior at room temperature, a property often described in terms of entropy. VOCs are commonly recognized as pollutants due to their adverse effects on air quality and environmental health. However, it is important to note that VOCs also play significant roles in biological interactions, such as communication between plants and animals. For instance, they serve as attractants for pollinators and contribute to inter-plant signaling^1^. In addition, some VOCs are dangerous to human health and cause harm to the environment. VOCs like acetone, n-butanol, toluene, styrene, and dimethyl disulfide are emitted from industrial sources (refineries, petrochemicals, paints, and pesticides, production plastics, etc) and they constitute about 7–10$\%$ of the whole atmospheric emissions^2^. The presence of volatile VOCs impacts not only the ecosystem but also the surrounding environment by contributing to the formation of greenhouse gases and the degradation of the stratospheric ozone layer. Additionally, VOCs pose hazards to human health, leading to irritation, nausea, and interference with the respiratory and nervous systems^3^.

The increasing stringency of environmental regulations, driven by the complexity of emissions from industries containing volatile organic compounds (VOCs), presents a growing concern. Among these industries, the paper and pulp sectors are notable emitters of methanol and $\alpha$-pinene. Methanol, classified as a hydrophobic chemical, has been identified by the United States Environmental Protection Agency as one of the most hazardous pollutants found in the air^4^. In contrast, $\alpha$-pinene is barely a water-soluble compound known as hydrophobic that can be found in softwoods. To avoid and overcome the causes of industries containing VOCs to the environment, the appropriate technologies have been proposed and developed to reduce emissions. These treatments are classified into chemical/physical systems (e.g. thermal incineration, wet scrubbing, chemical scrubbers, ozone oxidation, and carbon absorption) and biological systems (bio trickling filtration, biofiltration, sludge diffusions, and biofiltration)^5^. Each of these methods has advantages and disadvantages. Still, the detailed comparison has shown that biological treatments are less costly, less intensive, involve mild process conditions, have no secondary waste generation, and are ecologically clean. Among them, biofiltration (BF) has been tested to be the most propitious tool for odoriferous compounds (VOCs) owing to its constricted nature and flexibility^6,7^.

Recently, biofiltration has been successfully applied to study the entropy of $\alpha$-pinene, and methanol from the contaminated stream of the air. It is studied that hydrophilic methanol is an efficiently biodegradable compound that can quickly suppress the growth of hydrophobic compounds^8^. The infiltration process of pure $\alpha$-pinene^9^, and pure methanol^10,11^ was studied by several researchers. In addition, the biofilters are used for the polycyclic aromatic hydrocarbons^12^, mass transfer analysis in dual continuum models^12^, removal of gas-phase methanol in a compost/biochar biofilter^13^, volatile ethanol utilizing sugar cane marc inoculated with Candida utilis^14^ and enzymes with reversible Michaelis-Menten kinetics^15^.

Various mathematical models are proposed for the biophysical models of the biofiltration of volatile organic molecules that are hydrophilic and hydrophobic in composition. These models are highly nonlinear, and generally, finding exact solutions for such models is difficult. Different numerical and analytical techniques such as an iterative method^16^, homotopy perturbation method (HPM)^17^, spectral Chebyshev Wavelets method (SCWM)^18^, Elzaki homotopy transformation perturbation method (EHTPM)^19^, Bernoulli Wavelets method (BWM)^20^, Adomian decomposition method (ADM)^21^, optimal homotopy analysis method (OHAM)^22^, new spline algorithm^23^, Green’s function and fixed point iteration approach^24^ are developed to find the approximate solution for different problems modeling the reaction and diffusion phenomena in catalyst, biocatalyst, enzymatic reaction, and removal of different VOCs. These techniques have their advantages and limitations over one another. Also, they are gradient-based and purely deterministic approaches. Besides these algorithms, in recent times some machine learning algorithms are also employed to study the various domains of biofilteration. For example Fang^25^ investigated the use of machine learning approaches, including multilinear regression (MLR), artificial neural network (NN), and random forest (RF), to predict the performance of stormwater biofilters in heavy metal removal and risk mitigation. Their study found that the random forest model exhibited greater robustness in predicting metal removal compared to MLRs and NNs. Serrao^26^ developed a hybrid model for wastewater treatment process control. Combining a mechanistic model with a data-driven approach, the hybrid model improved accuracy by integrating information on poorly described sub-processes. Results showed significant performance enhancement compared to the mechanistic model alone, promising better control efficiency in wastewater treatment plants. Khan^15^ analyzed a mathematical model for a micro-disk biosensor using Michaelis-Menten kinetics. They transformed the reaction model into differential equations and employed a neural network with a Levenberg-Marquardt training algorithm for computational enhancement. In another study^27^ investigated the absorption of carbon dioxide (CO2) into phenyl glycidyl ether (PGE) solution, employing a machine learning approach. They utilized a nonlinear autoregressive exogenous (NARX) neural network model with two activation functions to calculate CO2 and PGE concentrations and flux based on reaction rate constants. Through supervised learning and training using a MATLAB solver, they validated their model’s results, demonstrating its rapid convergence, smooth implementation, and computational efficiency. The ML techniques are effectively employed for different studies such as penetration of both oil and water throughout the course of the secondary oil treatment procedure^28,29^, the Oldroyd coating to wire making prcoess^30^, Johnson Segalman flow of Fluid over an infinitely long vertical cylinder^31,32^, chaotic behaviour in wireless communication^33^, and nonlinear problems arising in heat transfer^34^. These applications motivate authors to explore the intellectual power of artificial neural networks by the use of an optimization approach to develop an alternate soft computing paradigm for addressing complex real-world problems. The important outcomes of this research are summarized as

- The mathematical models for biofiltration of VOCs such as hydrophilic (methanol) and hydrophobic ($\alpha$-pinene) along with first and zero-order kinetics are derived and analyzed by developing a novel design of intelligent computing integrated by Elman neural networks and Levenberg-Marquardt (LM) algorithm.
- The impact that shifts in a variety of parameters have on the entropy and saturation of methanol and $\alpha$-pinene alongside the removal ratio has been explored.
- The results calculated by the intended technique for various cases and scenarios are compared with Adomain decomposition method, Bernstein polynomial method, Chebyshev wavelets method, and solutions in terms of the fourth order of the Runge-Kutta technique. The design algorithm is executed multiple times to exhibit stability and accuracy by studying the solutions’ absolute errors and mean absolute deviations in each run.

## Mathematical formulation

**Figure 1:**
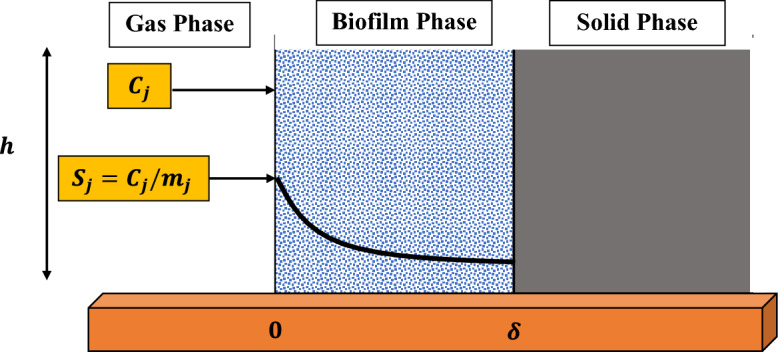
Structure of biofilm model with biofilter packing material.

**Figure 2:**
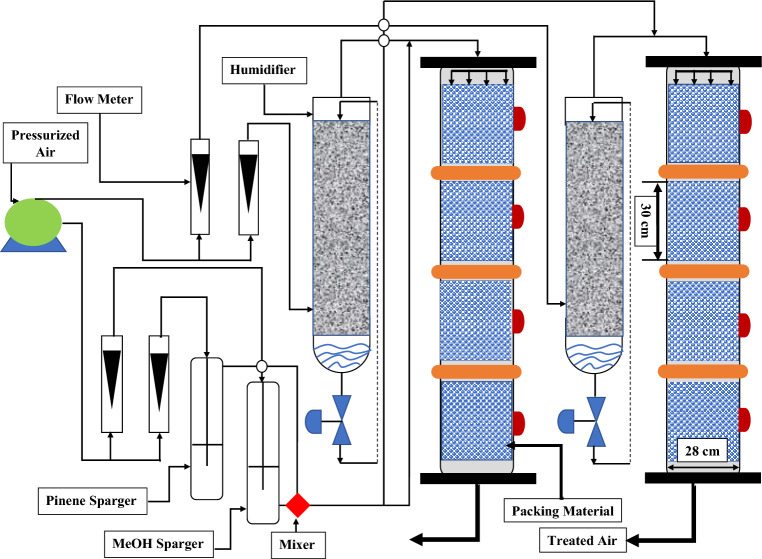
A graphical representation of the apparatus used in the experiment to carry out the biofiltration procedure.

The biophysical model of a single volatile organic compound that was presented by Van den Oever and Ottengraf in 1983 aids as the cornerstone for the mathematical paradigm of biofiltration for mixing volatile organic compounds (VOCs), such as hydrophilic and hydrophobic VOCs. The process includes two main components that are compound diffusion across biofilms and breakdown in the carriage of microorganisms. A conventional schema representation of an individual fragment in the biofilter that is coated by a homogeneous layer of biofilm and that is undergoing the simultaneous biodegradation of $\alpha$-pinene and methanol can be found in the Fig. 1. The experimental setup is given through Fig. 2. The derivation is based on the following assumptions:

- Across the biofilter the gradient of radial concentration is neglected, and airflow mimics the plug of the flow model.
- The biofilm grows over the exterior face of the particle. This is the proliferation of microorganisms on the surface of pores rather then inside. This demonstrates that there is no biodegradation in the pores.
- The biofilm completely occupies the padding media and has a very miniature thickness therefore, a rectangular design can be used.
- $\alpha$-pinene and methanol are the sole substrates that effects the biodegradation.
- The boundary layer of the gas phase is absent at the interface of air, hence the transformation of mass is ignored.
- Because the buildup of the biomass is anticipated to be slow under steady-state circumstances, it is believed that the film density will remain the same during the duration of the experiment.
- The microbial communities degrading $\alpha$-pinene, and methanol are different. Further details regarding the experimental setup can be found in^35^.

### Mass balance in the bio film phase

The structure of non-linear differential equations that describes the elimination of methanol and $\alpha$-pinene in biofilm at equilibrium state conditions are specified as

1 $$\begin{aligned}{} & {} D_{e m} \frac{d^{2} S_{m}}{d x^{2}}-\frac{X}{Y_{m}} \frac{\mu _{\max (m)} S_{m}}{\left( S_{m}+K_{m}\right) }=0, \end{aligned}$$

2 $$\begin{aligned}{} & {} D_{e p} \frac{d^{2} S_{p}}{d x^{2}}-\alpha \frac{X}{Y_{p}} \frac{\mu _{\max (p)} S_{p}}{\left( S_{p}+K_{p}\right) }=0, \end{aligned}$$

here, $S_m$ and $S_p$ are the saturations of methanol and $\alpha$-pinene respectively. *K* is constant of half saturation, *Y* is yield coefficient, $\mu _{max}$ denotes the rate of maximum specific growth, $D_{em}$ and $D_{ep}$ are the coefficients of effective diffusions of methanol and $\alpha$-pinene. *x* is the total population of microorganisms. $\alpha$ is the empirical constant that shows the influence of methanol on $\alpha$-pinene during the biodegradation which is given as

3 $$\begin{aligned} \alpha =\frac{1}{\left( 1+\left( \frac{C_{m}}{K_{i}}\right) ^{2}\right) }, \end{aligned}$$

In air phase, the entropy and saturation of methanol are denoted by $C_m$, $K_i$ is the inhibition constant. The boundary conditions subjected to Eqs. (1) and (2) are

4 $$\begin{aligned} \text { at } x= & {} 0, \quad S_{m}=\frac{C_{m}}{m_{m}}=S_{im } \text{ and } S_{P}=\frac{C_{P}}{m_{P}}=S_{ip}, \end{aligned}$$

5 $$\begin{aligned} \text { at } x= & {} \delta , \quad \frac{d S_{m}}{d x}=\frac{d S_{P}}{d x}=0. \end{aligned}$$

here, the $m_{m}$ and $m_{p}$ represent the mass of microorganisms associated with methanol and $\alpha$-pinene respectively, in the biofilm phase. These masses denote the quantity of microorganisms capable of metabolizing or interacting with the respective compounds. The following dimensionless parameters are defined to reduce Eqs. (1) and (2) into dimensionless mass balance equations

6 $$\begin{aligned} \beta= & {} \frac{S_{im}}{K_{m}}, \quad \phi =\frac{X \mu _{\max (m)}}{Y_{m}} \frac{\delta ^{2}}{D_{e m}K_{m}}, \quad S_{m}^{*}=\frac{S_{m}}{S_{im}}, \end{aligned}$$

7 $$\begin{aligned} \beta _{1}= & {} \frac{S_{i p}}{K_{p}}, \quad \phi _{1}=\frac{X \mu _{\max (p)}}{Y_{p}} \frac{\delta ^{2}}{D_{e p} K_{p}}, \quad S_{p}^{*}=\frac{S_{p}}{S_{i p}} \end{aligned}$$

Using the above dimensionless parameters in Eqs. (1) and (2) will result in

8 $$\begin{aligned}{} & {} \frac{d^{2} S_{m}^{*}}{d X^{* 2}}-\phi \left( \frac{S_{m}^{*}}{1+\beta S_{m}^{*}}\right) =0, \end{aligned}$$

9 $$\begin{aligned}{} & {} \frac{d^{2} S_{p}^{*}}{d X^{* 2}}-\alpha \phi _{1}\left( \frac{S_{p}^{*}}{1+\beta _{1} S_{p}^{*}}\right) =0, \end{aligned}$$

The boundary conditions are expressed as

10 $$\begin{aligned} \text{ at } \textrm{X}^{*}= & {} 0 \quad \mathrm {~S}_{p}^{*}=1 \quad \textrm{S}_{m}^{*}=1, \end{aligned}$$

11 $$\begin{aligned} \text{ at } X^{*}= & {} 1 \quad \frac{d S_{m}^{*}}{d X^{*}}=\frac{d S_{p}^{*}}{d X^{*}}=0. \end{aligned}$$

### Mass balance in gas phase

The concentration (saturation) of methanol and $\alpha$-pinene in biofilter along the air are governed by the nonlinear equation given as

12 $$\begin{aligned}{} & {} U_{g} \frac{d C_{m}}{d h}-A_{s} D_{e m}\left[ \frac{d S_{m}}{d x}\right] _{x=0}=0, \end{aligned}$$

13 $$\begin{aligned}{} & {} {U}_{g} \frac{d C_{p}}{d h}-A_{s} D_{e p}\left[ \frac{d S_{m p}}{d x}\right] _{x=0}=0, \end{aligned}$$

here, $C_p$ denotes the saturations of $\alpha$-pinene, $C_m$, represents the saturations of methanol in the gas/air phase. $U_g$ represents the gas velocity, $A_s$ is the area of a surface, and *h* denotes the status along the height of biofilters. The primary conditions subjected to Eqs. (12) and (13) are

14 $$\begin{aligned} \text { at } h=0, \quad C_{p}=C_{p i} \text{ and } C_{m}=C_{m i}. \end{aligned}$$

The following dimensionless parameters are defined to reduce Eqs. (12) and (13) into a non-dimensional mass balance equation in the gas patch.

15 $$\begin{aligned} \textrm{A}=\frac{HA_{s} D_{em} S_{im}}{U_{g} \delta C_{mi}},\quad A_{1}=\frac{HA_{s} D_{ep} S_{ip}}{U_{g} \delta C_{pi}}, \quad h^{*}=\frac{h}{H}, \quad C_{m}^{*}=\frac{C_{m}}{C_{mi}}, \quad C_{p}^{*}=\frac{C_{p}}{C_{p mi}}, \end{aligned}$$

Using the above dimensionless parameters in Eqs. (14) and (15) will result in

16 $$\begin{aligned}{} & {} \frac{d C_{m}^{*}}{d h^{*}}+A\left( \frac{d S_{m}^{*}}{d X^{*}}\right) _{X^{*}=0} = 0, \end{aligned}$$

17 $$\begin{aligned}{} & {} \frac{d C_{m p}^{*}}{d h^{*}}+\alpha A_{1}\left( \frac{d S_{p}^{*}}{d X^{*}}\right) _{X^{*}=0}=0, \end{aligned}$$

with conditions

18 $$\begin{aligned} C_{p}^{*}=\frac{C_{m p}}{C_{p i}} \text{ and } C_{m}^{*}=\frac{C_{m}}{C_{m i}} \text{ at } h^{*}=0. \end{aligned}$$

### Unsaturated kinetics

In this section, we review the case when the saturation of VOCs (methanol and $\alpha$-pinene) are relatively low in the biofilm juncture. In this study, $S_{m} \le K_{m}$ and $S_{p} \le K_{p}$, therefore, Eqs. (1) and (2) are reduced to

19 $$\begin{aligned}{} & {} D_{e m} \frac{d^{2} S_{m}}{d x^{2}}-\frac{X \mu _{\max (m)}}{Y_{m} K_{m}} S_{m}=0, \end{aligned}$$

20 $$\begin{aligned}{} & {} D_{e p} \frac{d^{2} S_{p}}{d x^{2}}-\alpha \frac{X \mu _{\max (p)}}{Y_{p} K_{p}} S_{p}=0, \end{aligned}$$

### Saturated kinetics

The mathematical model for biofiltration is considered when the saturation of methanol and $\alpha$-pinene is comparatively high. In this case, $S_{m} \ge K_{m}$ and $S_{p} \ge K_{p}$, therefore, Eqs. (1) and (2) are reduced to

21 $$\begin{aligned}{} & {} D_{e m} \frac{d^{2} S_{m}}{d x^{2}}-\frac{X}{Y_{m}} \mu _{\max (m)}=0, \end{aligned}$$

22 $$\begin{aligned}{} & {} D_{e p} \frac{d^{2} S_{p}}{d x^{2}}-\alpha \frac{X}{Y_{p}} \mu _{\max (p)}=0. \end{aligned}$$

## Proposed methodology

### Elman neural networks

**Figure 3:**
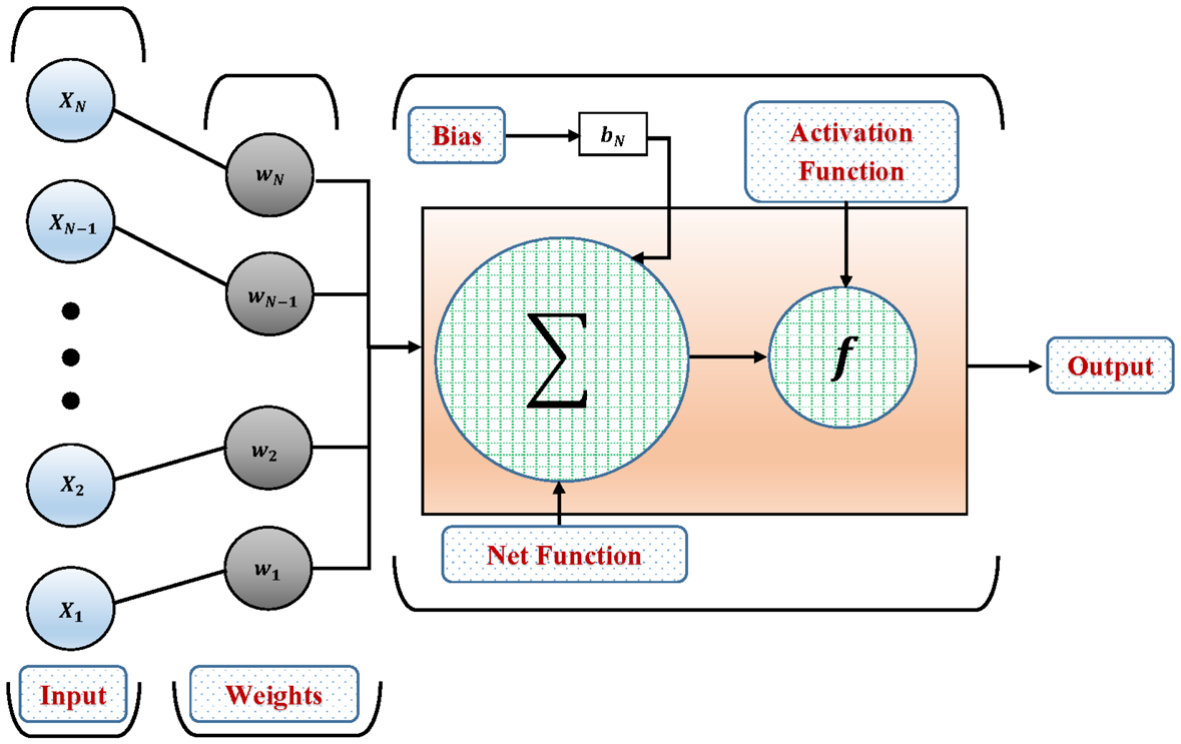
Details of a neuron in MLP network.

An artificial neural network (ANN), widely known as a neural network, is a computational model that mimics the behavior of biological brain. It comprises one input layer, one or more hidden layers, and an output layer. These layers are interconnected, with the input layer receiving data and passing it to deeper layers, which in turn forward signals to the output layer through the activation function. The information transmitted between layers undergoes multiple modifications before reaching the units responsible for constructing the inner levels, which are all hidden^36–38^. This architecture enables each layer to serve as both an input and output in solving complex problems. Figure 3 illustrates the framework and layout of an ANN.

**Figure 4:**
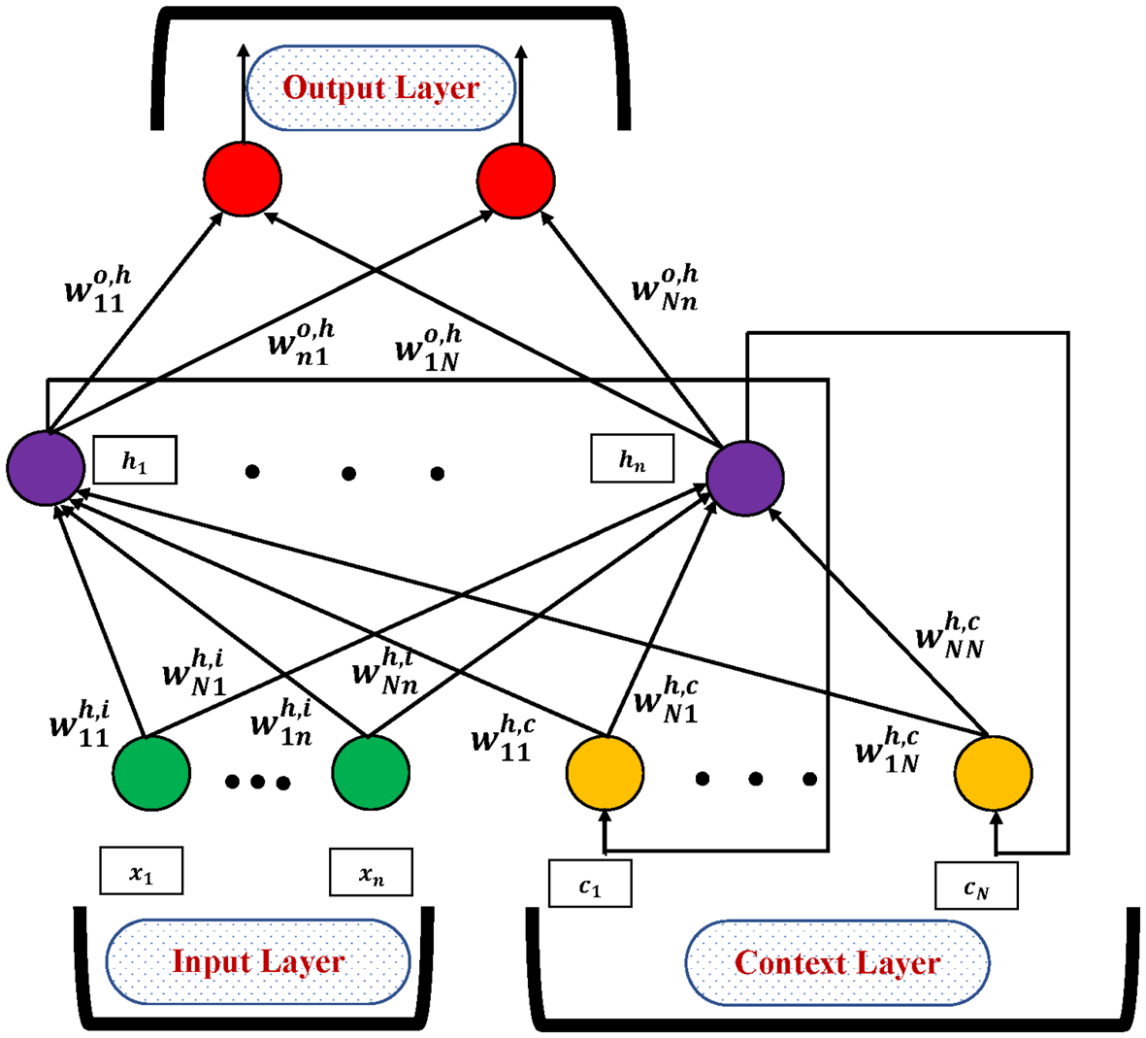
The internal organization of the Elman neural network.

An Elman neural network (ENN) is a three-layer structure with an additional context layer. Figure 4 illustrates the connection of these hidden neurons to the context layers. Besides, the traditional three layers, the context layer accepts the input from the output of the hidden layer and maintains the state of the preceding time’s values^39,40^.

We consider the architecture of the Elman neural network as depicted in Fig. 4, with output weight matrix, context, and external input layers which are denoted by $w^{o, h}(t), w^{h, c}(t), w^{h, i}(t)$. Also, the n-dimensional input and output vectors are $x^{1}(t)=\left[ x_{1}^{1}(t), x_{2}^{1}(t), \ldots , x_{n}^{1}(t)\right] ^{T}$ and $y(t)=\left\lfloor y_{1}(t), y_{2}(t), \ldots , y_{n}(t)\right\rfloor ^{T}$. Additionally, the count of hidden neurons is N therefore, $w^{h, i}(t) \in R^{N \times n}$, $w^{h, c}(t) \in R^{N \times N}$, and $w^{o, h}(t) \in R^{n \times N}$. The output vector of the hidden layer $c(t-1)=\left[ c_{1}(t-1), c_{2}(t-1), \ldots , c_{m}(t-1)\right] ^{T}$, is associated back to the hidden layer as another input vector so $x^{2}(t)=\left[ x_{1}^{2}(t), x_{2}^{2}(t), \ldots , x_{N}^{2}(t)\right] ^{T}=c(t-1)$, so the entire input vector is defined as

23 $$\begin{aligned} \begin{aligned} x(t)&=\left[ x_{1}^{1}(t), x_{2}^{1}(t), \ldots , x_{n}^{1}(t), x_{n+1}^{2}(t), \cdots , x_{k}^{2}(t)\right] ^{T}, \\&=\left[ \left[ x^{1}(t)\right] ^{T}\left[ x^{2}(t)\right] ^{T}\right] ^{T}, \\&=\left[ x_{1}^{1}(t), x_{2}^{1}(t), \ldots , x_{n}^{1}(t), c_{1}^{2}(t-1), \cdots , c_{N}^{2}(t-1)\right] ^{T}, \end{aligned} \end{aligned}$$

therefore, The output vector may be determined by using the given equation

24 $$\begin{aligned} y_{i}(t)=f\left( a_{i}^{o}(t)\right) =\frac{1}{1+\exp \left( -a_{i}^{o}(t)\right) }, i=1,2, \ldots , n \end{aligned}$$

where, *f* is the activation function and $a_{i}^{o}(t)$ is given as

25 $$\begin{aligned} a_{i}^{o}(t)=\sum _{j=1}^{N} W_{j i}^{o, h}(t) * h_{j}(t), i=1,2, \ldots , n. \end{aligned}$$

### Optimization and training of neurons

**Figure 5:**
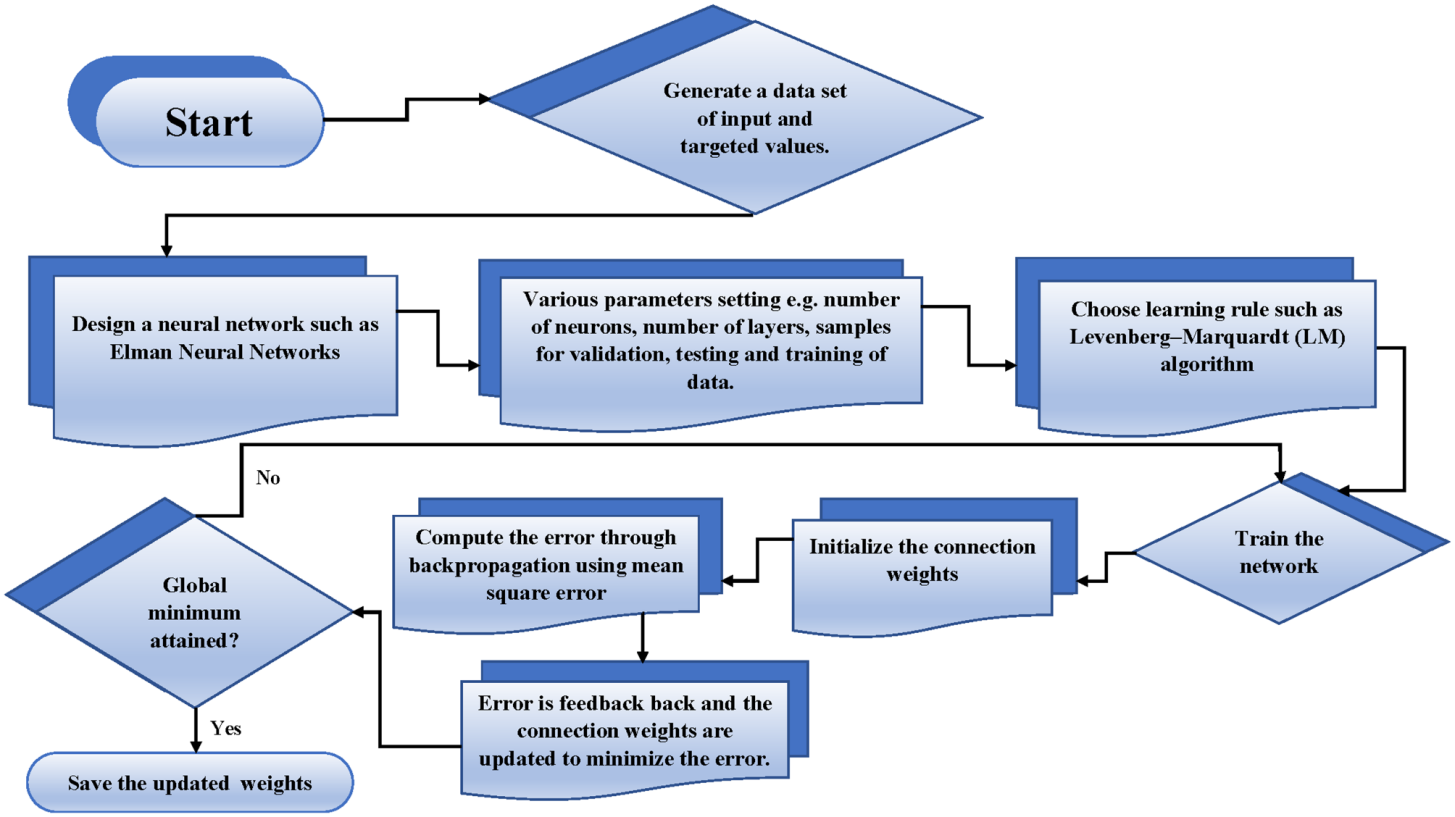
The operational mechanism of the design algorithm carried out in a sequential fashion.

This section describe the training that is required for the unknown weights/ neurons that are included in the ENN framework. The neurons involved in ENN are adjusted using the optimization-based local search technique known as the Levenberg-Marquardt (LM) algorithm. LM algorithm is a curve fitting least square technique that is used for the minimization problems and it converges faster then order training algorithms^41^. Among the more recent implementations and uses of the LM algorithm are the identification of Hammerstein nonlinear systems^42,43^, predicting drag reduction in crude oil pipelines^44^, charging state in the batteries with lithium-ions^45,46^, blind source separation of joint diagonalization^47^ and parameter estimation of inverse heat transfer problems^48^.

Moreover, the actualization of the design algorithm consists of two distinct stages. Initially, an Adam’s method is utilized to generate a dataset or targeted data comprising 1001 points within the range of [0,1] for various cases of mathematical models representing the saturation of methanol and $\alpha$-pinene in the biofilm and air phases. This dataset is divided into three subsets: a training set, a validation set, and a test set, typically in proportions such as 70%, 15%, and 15%, respectively. In the second phase, the Elman neural network is configured with initial parameter settings as provided in Table 1. The parameters chosen in Table 1 were determined based on a combination of factors, including prior knowledge of similar neural network architectures used in related studies^49–51^, computational resources available, and practical considerations regarding model complexity and training time. While an optimization strategy was not explicitly employed to define the number of layers and neurons in this specific instance, the selection process involved experimentation and iterative refinement. We conducted preliminary trials with different configurations of layers and neurons and assessed their performance using metrics such as mean square error on validation datasets. The chosen configuration of 2 layers with 60 neurons was found to provide a balance between model complexity and predictive accuracy within the constraints of our study. Subsequently, an appropriate learning algorithm, such as the Levenberg-Marquardt (LM) optimization paradigm, is implemented to achieve optimal weighting inside the ENN framework and validate the estimated solutions for the problem. An overview and workflow of the designed scheme are depicted in Fig. 5.

**Table 1:** Essential parameter setting for the accomplishment of the proposed ENN-LMA scheme.

Parameter	Settings	Parameter	Settings
Number of neurons	60	Layers	2
Performance function	Mean Square error	Maximum iterations	2000

## Numerical experimentation and discussion

**Table 2:** Comparative analysis of the approximate solutions for the saturation of methanol in biofilm phase, acquired by the suggested ENN-LM scheme with RK-4, CWM^8^, and ADM^52^.

$X^{*}$	$\phi = \beta =10$				$\phi =10, \beta =15$				$\phi =10, \beta =300$			
	RK-4	ADM	CWM	ENN-LM	RK-4	ADM	CWM	ENN-LM	RK-4	ADM	CWM	ENN-LM
0	1	1	1	0.999996	1	1	1	0.999936	1	1	0.9999	1
0.2	0.843313	0.836364	0.8381	0.843315	0.889549	0.8875	0.8879	0.889503	0.99402	0.99402	0.9939	0.99402
0.4	0.722383	0.709091	0.7132	0.722384	0.803907	0.8000	0.8040	0.803911	0.989369	0.989369	0.9889	0.989369
0.6	0.636594	0.618182	0.6174	0.636490	0.742891	0.7375	0.7420	0.742891	0.986047	0.986047	0.9861	0.986047
0.8	0.585389	0.563636	0.5700	0.585381	0.706351	0.7000	0.7050	0.706355	0.984054	0.984053	0.9838	0.984054
1	0.568368	0.545455	0.5520	0.568382	0.694182	0.6875	0.6934	0.694129	0.983389	0.983389	0.9829	0.983389

**Table 3:** Comparison of approximate solutions for the saturation of $\alpha$-pinene in biofilm phase, acquired by the suggested ENN-LM scheme with RK-4, BPM, CWM^8^, and ADM^52^.

$X^{*}$	$\alpha = 1, \beta = 10, \phi = 0.1$					$\alpha = 1, \beta = 10, \phi = 4$				
	RK-4	CWM	BPM	ADM	ENN-LM	RK-4	CWM	BPM	ADM	ENN-LM
0	1	1	1	1	1	1	1	1	1	
0.2	0.998364	0.9983	0.9983	0.998364	0.998364	0.935439	0.9345	0.9344	0.934545	0.935433
0.4	0.997092	0.997	0.997	0.997091	0.997092	0.885333	0.8833	0.8833	0.883636	0.885345
0.6	0.996183	0.9961	0.9961	0.996182	0.996183	0.849605	0.8475	0.849	0.847273	0.849612
0.8	0.995638	0.9956	0.9956	0.995636	0.995638	0.828194	0.8252	0.8286	0.825455	0.828192
1	0.995456	0.9954	0.9954	0.995455	0.995456	0.821062	0.8183	0.8285	0.818182	0.821056

In this section, the design technique of the ENN-LM algorithm is implemented on the mathematical model for biofiltration of methanol and $\alpha$-pinene in biofilm and air phases. The influence that changes in different parameters had on concentration profiles is investigated. The estimated solutions by ENN-LM algorithm for saturation of methanol and $\alpha$-pinene in biofilm juncture are in contrast to the numerical values secured by the use of the Runge-Kutta technique (RK-4), Bernstein polynomial method (BPM), Chebyshev wavelets method (CWM)^8^, and Adomian decomposition method (ADM)^52^ as shown in Tables 2 and 3. The results demonstrate the findings obtained from the design algorithm overlap the results of the numerical calculations and have the least amount of absolute error when correlated to other state-of-the-art methodologies.

**Figure 6:**
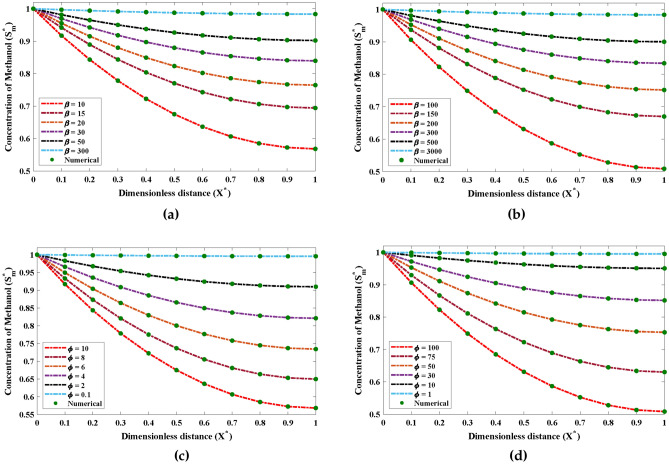
The potential impact of the variances in $\beta$ and $\phi$ on the saturation profile of methanol in biofilm phase.

Figure 6 demonstrates the impact that various values have on $\alpha$ and $\beta$ on dimensionless saturation profile. It can be inferred from Fig. 6a and b that the saturation of methanol during the biofiltration process increases with the growth in the initial saturation of methanol $\beta$. It is worth noticing that for large values of $\beta$ the saturation remains steady. The influence of variations in $\phi$ with fixed value of $\alpha$ on saturation of methanol is shown through Fig. 6c and d. It can be noticed that maximum growth rate of methanol biodegradation $\phi$ causes decrease in the saturation and finally becomes firm at higher values.

**Figure 7:**
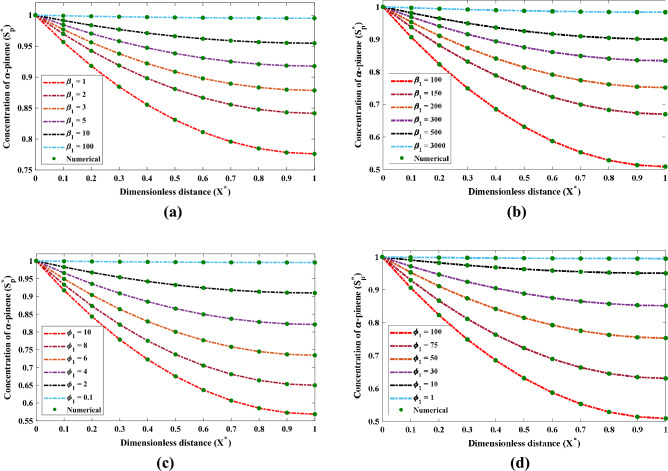
The effect of differences in $\beta _1$ and $\phi _1$ on the saturation profile of $\alpha$-pinene in biofilm phase.

The result of several factors such as $\beta _1$ and $\phi _1$ on the dimensionless saturation of $\alpha$-pinene are shown through Fig. 7. It is evident from the following Fig. 7a and b that the saturation (saturation) rises with enhancement in $\beta _1$ and fixed values of dry cell density. Also, the increase in saturation is observed when the denseness of the film reduces.

**Figure 8:**
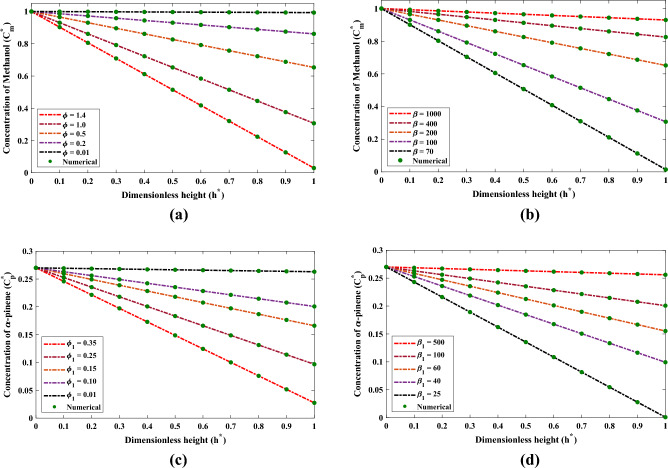
The influence that changes in the various factors have on (**a**, **b**) saturation drafts of methanol and (**c**, **d**) $\alpha$-pinene during the biofiltration process at air phase.

In addition, the saturation profiles of methanol and $\alpha$-pinene under the sway of alterations in $\beta$, $\beta _1$, $\phi$ and $\phi _1$ in air phase are shown though Fig. 8. The saturation of methanol increases and gradually reaches to the stable state (constant) when the film denseness or $\phi$ increases. Also, with a decrease in half saturation parameter, the saturation decreases. The conclusion that can be drawn from Fig. 8c and d is that the constant level of saturation of $\alpha$-pinene is achieved after an increase with the increase in diffusion coefficient and initial saturation parameter with fixed values of $\alpha$.

**Figure 9:**
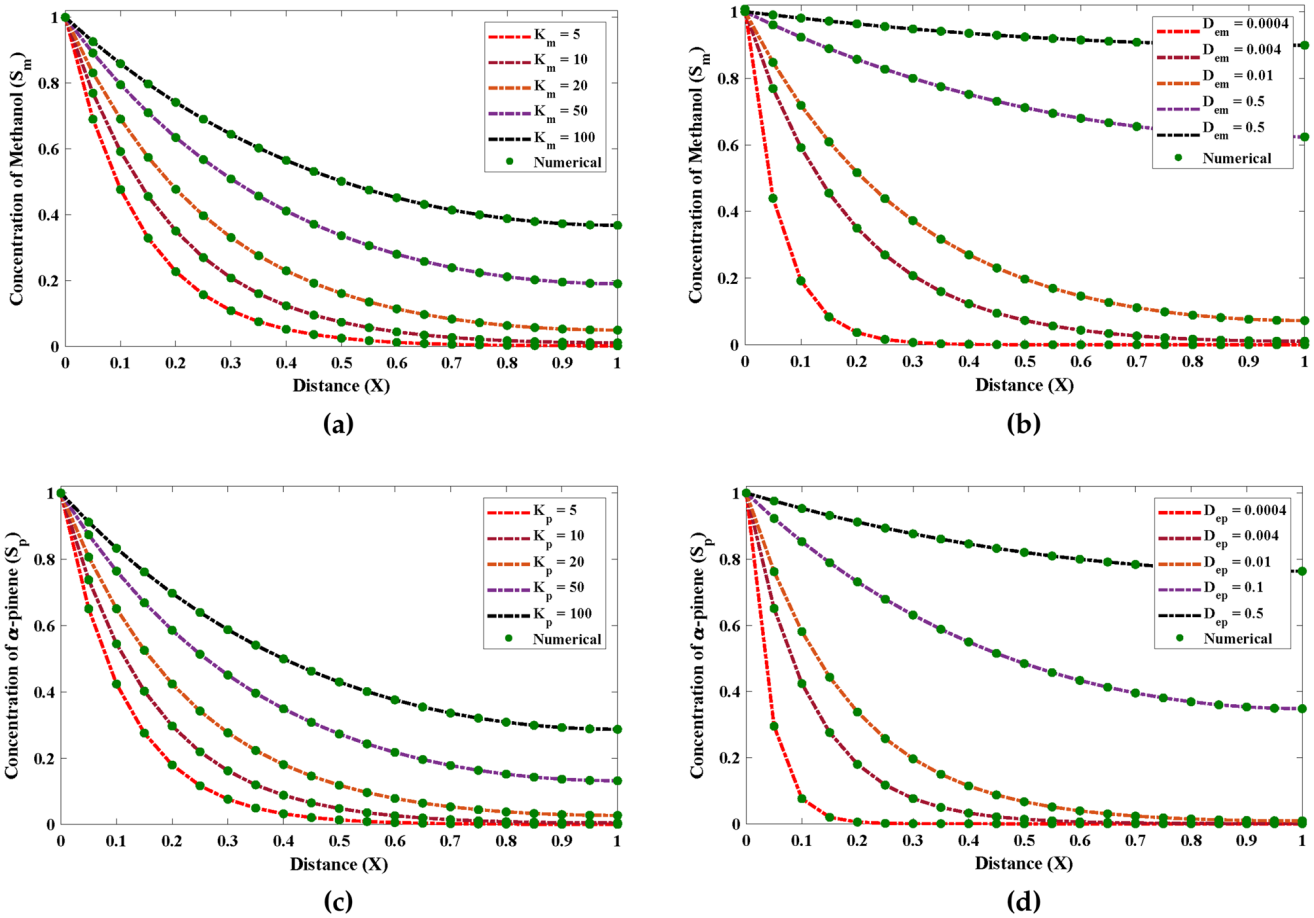
The impact of changes in the values of the various parameters on the saturation of methanol and $\alpha$-pinene for unsaturated (first order) kinetics.

**Figure 10:**
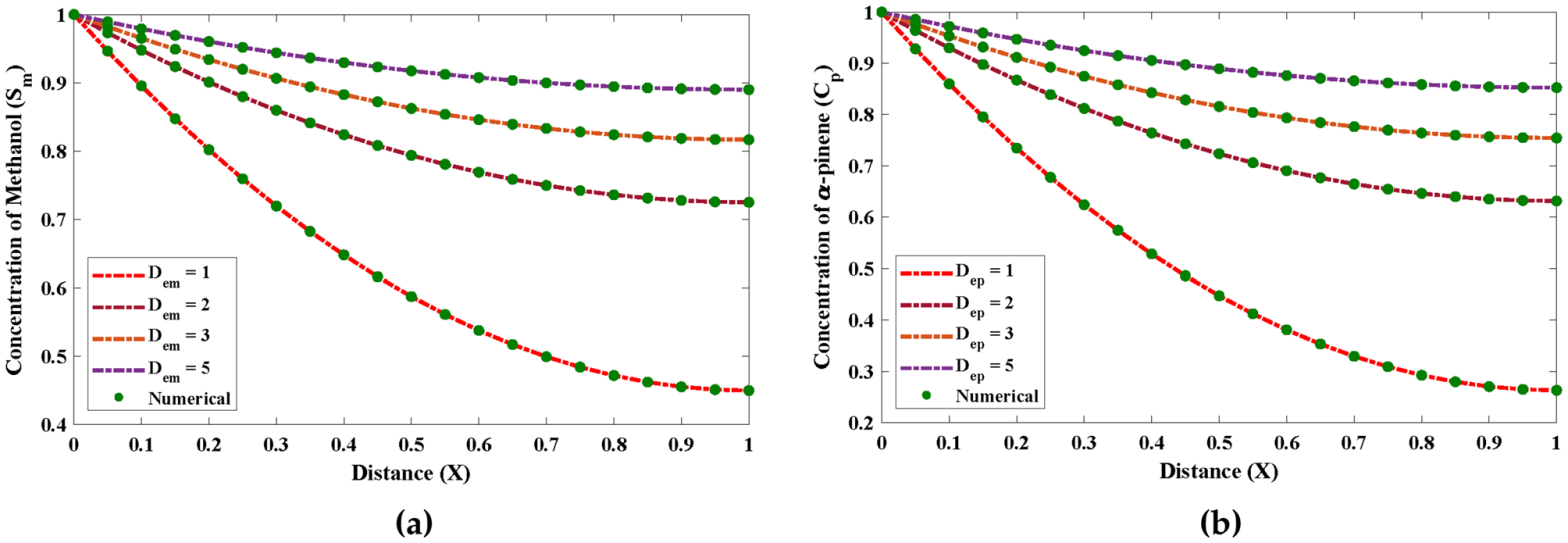
The consequence of deviations in the values of the various parameters on the saturation of methanol and $\alpha$-pinene for saturated (zero order) kinetics.

Further, the suggested technique is implanted to investigate how specific parameters affect the unsaturated and saturated kinetic of the saturation of methanol and $\alpha$-pinene during the biofilteration. Each parameter is changed while the others, which are thought to be constants are given as $D_{em} = D_{ep} = 0.004, \frac{X \mu _{max(m)}}{Y_m}=1.1, \alpha = 1.34$ and $K_m = K_p = 10$. The results of saturation profiles obtained by the ENN-LM algorithm by varying different parameters are illustrated through Figs. 9 and 10. The approximate and numerical solutions overlap each other, which demonstrates the precision and robustness of the recommended design technique in approximating stiff nonlinear problems.

**Figure 11:**
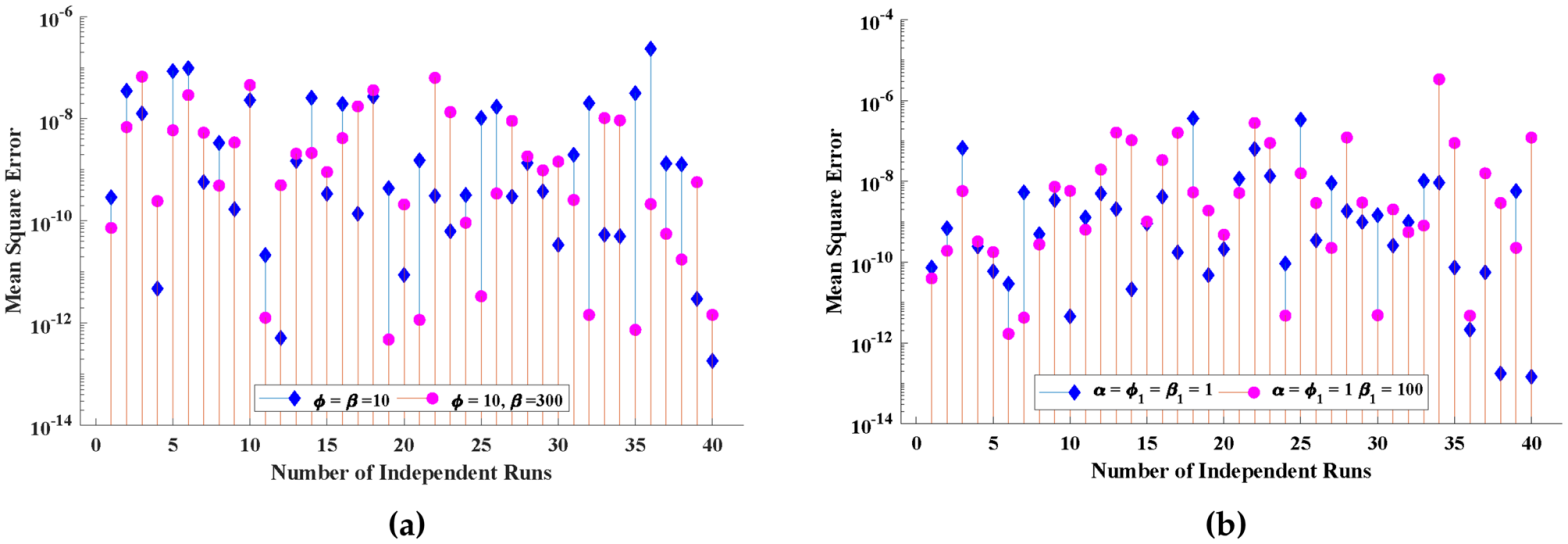
Performance values in term of mean square error obtained by the proposed algorithm during multiple execution.

For the purpose of conducting a comprehensive analysis of the intended ENN-LM method, the number of independent execution of the design scheme is carried out to beget huge data for the statistical and numerical analysis to exhibit the solutions’ stability, efficiency, and accuracy. In this regard, different performance indexes are defined in terms of solutions deviation (MAD), coefficient of Theil’s inequality (TIC), and error in Nash-Sutcliffe efficiency (ENSE). These performance metrics are formulated mathematically as follows:

26 $$\begin{aligned} \left[ M A D_{S_m^{*}}, M A D_{S_p^{*}}\right]= & {} \left[ \begin{array}{l} \frac{1}{M} \sum _{j=1}^{M}\left| \bar{S_m^{*}}\left( X_{j}\right) -S_m^{*}\left( X_{j}\right) \right| ,\\ \frac{1}{M} \sum _{j=1}^{M}\left| \bar{S_p^{*}}\left( X_{j}\right) -S_p^{*}\left( X_{j}\right) \right| , \end{array}\right] ^{t}, \end{aligned}$$

27 $$\begin{aligned} \left[ \text{ TIC } _{S_m^{*}}, \text{ TIC } _{S_p^{*}}\right]= & {} \left[ \begin{array}{c} \frac{\sqrt{\frac{1}{M} \sum _{j=1}^{M}\left( \bar{S_m^{*}}\left( X_{j}\right) -S_m^{*}\left( X_{j}\right) \right) ^{2}}}{\sqrt{\frac{1}{M} \sum _{j=1}^{M}\left( \bar{S_m^{*}}\left( X_{j}\right) \right) ^{2}}+\sqrt{\frac{1}{M} \sum _{j=1}^{M}\left( S_m^{*}\left( X_{j}\right) \right) ^{2}}}, \\ \frac{\sqrt{\frac{1}{M} \sum _{j=1}^{M}\left( \bar{S_p^{*}}\left( X_{j}\right) -S_p^{*}\left( X_{j}\right) \right) ^{2}}}{\sqrt{\frac{1}{M} \sum _{j=1}^{M}\left( \bar{S_p^{*}}\left( X_{j}\right) \right) ^{2}}+\sqrt{\frac{1}{M} \sum _{j=1}^{M}\left( S_p^{*}\left( X_{j}\right) \right) ^{2}}}, \end{array}\right] ^{t}, \end{aligned}$$

28 $$\begin{aligned} \left[ N S E_{S_m^{*}}, N S E_{S_p^{*}}\right]= & {} \left[ \begin{array}{cc} 1-\frac{\frac{1}{M} \sum _{j=1}^{M}\left( \bar{S_m^{*}}\left( X_{j}\right) -S_m^{*}\left( X_{j}\right) \right) ^{2}}{\sum _{j=1}^{M}\left( \bar{S_m^{*}}\left( X_{j}\right) -\widehat{S_m^{*}}\left( X_{j}\right) \right) ^{2}}, &{} \widehat{S_m^{*}}\left( X_{j}\right) =\frac{1}{M} \sum _{j=1}^{M} S_m^{*}\left( X_{j}\right) , \\ 1-\frac{\frac{1}{M} \sum _{j=1}^{M}\left( \bar{S_p^{*}}\left( X_{j}\right) -S_p^{*}\left( X_{j}\right) \right) ^{2}}{\sum _{j=1}^{M}\left( \bar{S_p^{*}}\left( X_{j}\right) -\widehat{S_p^{*}}\left( X_{j}\right) \right) ^{2}}, &{} \widehat{S_p^{*}}\left( X_{j}\right) =\frac{1}{M} \sum _{j=1}^{M} S_p^{*}\left( X_{j}\right) , \\ \end{array}\right] ^t \end{aligned}$$

29 $$\begin{aligned} \left[ E N S E_{S_m^{*}}, E N S E_{S_p^{*}}\right]= & {} \left[ 1-N S E_{S_m^{*}}, 1-N S E_{S_p^{*}}\right] . \end{aligned}$$

here, *M* shows the number of independent executions, $\bar{S_m^{*}}$, $\bar{S_p^{*}}$, $S_m^{*}$ and $S_p^{*}$ are the analytical and approximate solutions for biofilm and gas phases respectively.

**Figure 12:**
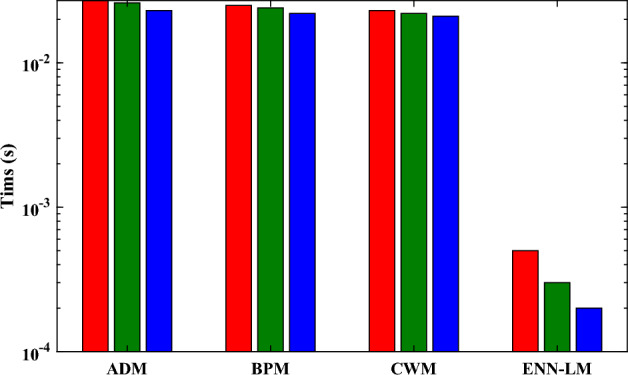
Illustration of the time required/taken by ADM, BPM, CWM and ENN-LM for computing an approximation of the saturation of methanol with $\phi = \beta =10$.

The objective values or performance values referring to the root mean square deviation is dictated through Fig. 11. It is worth noticing that the values of MSE are approaching to zero for different case of biofilm and air phases during the biofiltration of VOCs. In addition, Table 4 displays the lowest and maximum values of performance metrics, together with their standard deviations during the multiple exsections of the ENN-LM algorithm. It can be observed that global (mean) values lies around $10^{-5}$ to $10^{-7}$ that reveals the exactness and robustness of the intended supervised layout. Figure 12 is plotted to exemplify the efficacy and potency of the deliberate approach in terms of the execution time. It is clear that the ENN-LM algorithm is much faster than the technique available in the latest literature.

**Table 4:** Statistical analysis based on minimum, average results and deviations of the performance indicators for cases in a mathematical model of biofilm and air phases during the biofiltration of VOCs.

	Mean absolute deviations			Theil’s Inequality coefficient			Error in Nash-Sutcliffe efficiency		
	Minimal	Average	Standard Div.	Minimal	Average	Standard Div.	Minimal	Average	Standard Div.
$\phi = \beta = 10$	3.2974809E−06	8.1490624E−05	7.3083689E−05	5.3495240E−07	1.2113816E−05	1.0704903E−05	1.2340207E−10	1.3446792E−07	2.3083275E−07
$\phi = 10, \beta = 300$	1.4234789E−06	6.3737440E−05	1.2303922E−04	2.0429463E−07	9.4166076E−06	1.8417880E−05	2.1468605E−11	1.9952532E−07	9.7876075E−07
$\phi _1 = \beta _1 = \alpha =1$	1.1879083E−06	7.4497471E−05	6.8402009E−05	1.8114836E−07	1.1270868E−05	1.0322667E−05	1.6014856E−11	1.1475828E−07	1.7689733E−07
$\phi _1 = \alpha _1=1, \beta _1 = 100$	2.2965465E−06	4.5612638E−05	5.5761701E−05	3.0255401E−07	6.6627934E−06	8.1987300E−06	5.5879523E−11	5.4163444E−08	1.3106060E−07

## Conclusion

In this article, we conduct an investigation of the mathematical models of biofiltration for mixing volatile organic compounds (VOCs) such as methanol and hydrophobic $\alpha$-pinene in the biofilm and air phases, respectively. The models are based on nonlinear diffusion equations. Furthermore, we have developed a machine learning artificial intelligence-based computing technique to study the saturation profiles of methanol and $\alpha$-pinene. Various parameters have been varied to study their effect on the saturation of saturated and unsaturated kinetics of the VOCs. The results demonstrate that methanol saturation during the biofiltration process increases with an increase in the initial saturation of methanol $\beta$ and maximum growth rate of methanol biodegradation $\phi$ causes a decrease in the saturation. Also, the saturation of $\alpha$-pinene increases with an increase in $\beta _1$ with fixed values of dry cell density. The results captured by the intended ENN-LM method are contrasted to the solutions of numerical studies by the Runge-Kutta method (RK-4), Bernstein polynomial method (BPM), Chebyshev wavelets method (CWM), and Adomian decomposition method (ADM) that illustrates the precision and effectiveness of the method that has been suggested. The results of mean square error, mean absolute divisions, and the importance of the design method are further shown by the complexity of the computations.

## Data Availability

The data that support the findings of this study are available on request from the corresponding author.
